# Mutations in the stator protein PomA affect switching of rotational direction in bacterial flagellar motor

**DOI:** 10.1038/s41598-022-06947-5

**Published:** 2022-02-22

**Authors:** Hiroyuki Terashima, Kiyoshiro Hori, Kunio Ihara, Michio Homma, Seiji Kojima

**Affiliations:** 1 Division of Biological Science, Graduate School of Science, Nagoya University, Furo-cho, Chikusa-ku, Nagoya, 464-8602 Japan; 2 Department of Bacteriology, Institute of Tropical Medicine (NEKKEN), Nagasaki University, 1-12-4 Sakamoto, Nagasaki, 852-8523 Japan; 3 Center for Gene Research, Nagoya University, Furo-cho, Chikusa-ku, Nagoya, 464-8602 Japan

**Keywords:** Biophysics, Microbiology, Molecular biology

## Abstract

The flagellar motor rotates bi-directionally in counter-clockwise (CCW) and clockwise (CW) directions. The motor consists of a stator and a rotor. Recent structural studies have revealed that the stator is composed of a pentameric ring of A subunits and a dimer axis of B subunits. Highly conserved charged and neighboring residues of the A subunit interacts with the rotor, generating torque through a gear-like mechanism. The rotational direction is controlled by chemotaxis signaling transmitted to the rotor, with less evidence for the stator being involved. In this study, we report novel mutations that affect the switching of the rotational direction at the putative interaction site of the stator to generate rotational force. Our results highlight an aspect of flagellar motor function that appropriate switching of the interaction states between the stator and rotor is critical for controlling the rotational direction.

## Introduction

Bacteria can swim in an aqueous environment or swarm on a surface through screw-like rotation of a flagellum. The flagellar motor consists of stator and rotor elements. The stator, which functions as an ion channel, conducts ions, such as H^+^ in *Escherichia coli* or Na^+^ in *Vibrio alginolyticus*, along a transmembrane electrochemical potential difference. The ion influx induces conformational changes in the stator, followed by changing the interaction between the stator and rotor to generate rotation power. Being bidirectional, the flagellar motor can rotate in either counter-clockwise (CCW) or clockwise (CW) direction. The rotational direction is associated with chemotaxis, the ability of a bacterium to sense the environment around itself and migrate towards a more suitable location and away from unfavorable stimuli^1,2^. The cells swim straight (forward) when the flagella rotate in CCW direction. Once the cells sense an unfavorable chemical or temperature, they change the direction of flagellar rotation from CCW to CW, resulting in changes in the direction of the cell body.

The stator is composed of two kinds of membrane proteins, MotA and MotB for *E. coli* or PomA and PomB for *V. alginolyticus*^3,4^. MotA and PomA are membrane proteins with four transmembrane segments (TM) and a large cytoplasmic region (Loop_2–3_) between TM2 and TM3. MotB and PomB are membrane proteins with a single TM and a periplasmic peptidoglycan-binding (PGB) domain in the C-terminal region. The atomic structures of the stator were determined by two different groups^5,6^. The MotA/MotB and PomA/PomB complexes, previously proposed as 4:2 hetero-hexamers, were shown to be 5:2 hetero-heptamers. From the structure in which two molecules of MotB are inserted in the center of the MotA five-molecule ring, a gear-like rotation model between the stator and rotor has been proposed^5–8^. In this model, the MotA ring may rotate in CCW direction by default with respect to the axis of MotB coupled to the influx of ions through a transmembrane ion channel.

**Figure 1:**
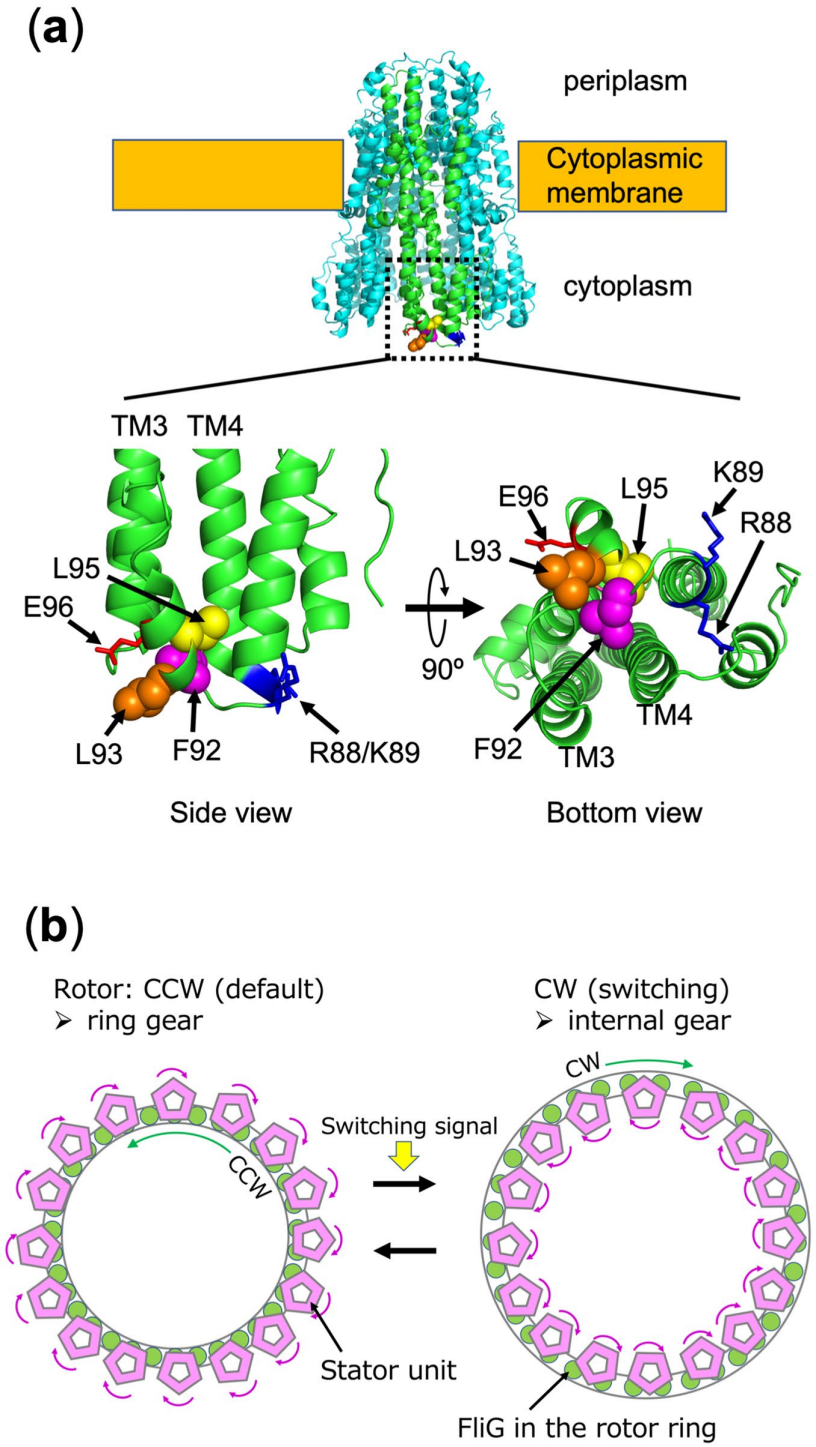
Model of an interaction surface of stator against rotor. (**a**) A whole ribbon model of *C. jejuni* MotA/B complex (6YKM) (upper image) and a enlarged part of the ribbon model of the *C. jejuni* MotA monomer (lower images of side and bottom views). These lower images were represented the cytoplasmic region (Loop_2–3_) of the MotA monomer. The corresponding residues in *V. alginolyticus* PomA are represented by arrows and residue numbers. (**b**) Switching model of flagellar motor rotation. The rotational force of stator is transmitted to the rotor through a gear-like motion. In the default, the rotor rotates with CCW direction engaged by the stator rotation. It has been hypothesized that the rotor interacts with the inner tooth of stator gear for CCW rotation and changes with the outer tooth of stator gear for CW rotation. When CheY-P binds the rotor to induce structural changes of rotor or C-ring, the rotor size expands for the CW structure by the swiching signal. The stator and rotor are shown in pink pentagons and in green circles, respectively.

The rotor is a huge ring complex composed of a transmembrane MS-ring formed by the transmembrane protein FliF and a cytoplasmic C-ring consisting of FliG, FliM and FliN^9–15^. The stator–rotor interaction arises between the C-terminal region of FliG and the cytoplasmic region of the stator A subunit to generate torque^16,17^. Previous genetic studies have shown that conserved charged residues in the A subunit (MotA R90 and E98 in *E. coli* and PomA R88 and E96 in *V. alginolyticus*) are associated with those in FliG (FliG R281 and D288 in *E. coli* and FliG R301 and D308 in *V. alginolyticus*) through electrostatic interactions^18–22^. Recently, we have provided the direct evidence for physical interactions between PomA and FliG biochemically by using site-directed in vivo photo-crosslinking and cysteine disulfide-crosslinking. Moreover, we have also identified that D85, K89, G90, F92, and L93 residues of PomA are located close to FliG (Fig. 1a, Fig. S1)^7^. The region containing these residues was located at the external and membrane-distal portion of the A subunit. This region is not directly involved in the ion conduction.

The direction of rotation is controlled by the C-ring (also called the switch complex). The chemotaxis signaling protein, CheY, is phosphorylated via intracellular signaling pathways from chemoreceptors^23,24^. The phosphorylated form of CheY (CheY-P) binds to a well-conserved motif in the N-terminal region of FliM (FliM_N_)^25–29^. CheY-P binding induces structural changes in the middle domain of FliM^30^. As a result, a structural change occurs at the FliG-stator interaction site, thereby switching the direction of rotation of the flagellar motor from CCW to CW^31^. After the dissociation of CheY-P from the C-ring, the rotational direction return to CCW. The number of stator units incorporating into the motor is dependent on the external mechanical loads^32,33^. Increase in the stator incorporation at the viscous load induces conformational shifts in FliM/N, resulting in modulating the binding affinity for CheY-P to the switch complex^34^. An increase in external load increases the binding affinity of the C-ring for CheY-P, suggesting stator–rotor interaction contributes to the switching of rotation direction of the flagellar motor. In *V. alginolyticus*, a single transmembrane protein called ZomB is also necessary for the CW rotation, depending on CheY^35^.

Cryo-electron tomography of intact flagellar motors in *Borrelia burgdorferi* and *V. alginolyticus* showed that the diameter of the C-ring expands during CW rotation^8,36^. The C-terminal region of FliG, at the top of the C-ring close to the membrane, appears to interact with the stator on its inner side facing the rotor axis in CCW rotation, whereas it appears to interact with the stator on its outer side away from the rotor axis in CW rotation (Fig. 1b). Therefore, the rotation direction of the flagellar motor is determined by changing the portion of the stator interacting with the rotor. Previous genetic studies have reported that mutations in the stator confer Mot^−^ phenotype (non-motile) in many cases. In some cases, mutations in the stator confer Che^−^ phenotype (no chemotaxis or inhibited switching of rotational direction)^37–39^. For example, MotA T21I/MotB A39V in *E. coli* confers CW-biased rotation and PomA R88A/K89A/E96Q/E97Q/E99Q in *V. alginolyticus* confers CCW-biased rotation. Therefore, the rotational direction is probably affected by conformational change in the stator or stator–rotor interface though it is mainly determined by the CheY-P binding or the conformational change of the C-ring.

In this study, we focused on the F92 and L93 residues of PomA that have been identified to be very close to FliG^7^. We also focused on the PomA-L95 residue, which is highly conserved and is located next to the conserved glutamate residue, an important charged residue involved in torque generation. The substitution of these three residues to a charged residue decreases the switching frequency of the motor and is biased toward CCW rotation. Furthermore, when combined with a CW-biased mutation in FliG, they inhibited the switching of the motor and stabilized the CW rotation, indicating that L93 and L95 of PomA are involved in efficient and robust switching of flagellar motor rotation, providing novel insights into the gear rotation model of the flagellar motor.

## Results

### Residues of PomA mutations are involved in stator–rotor interaction

**Figure 2:**
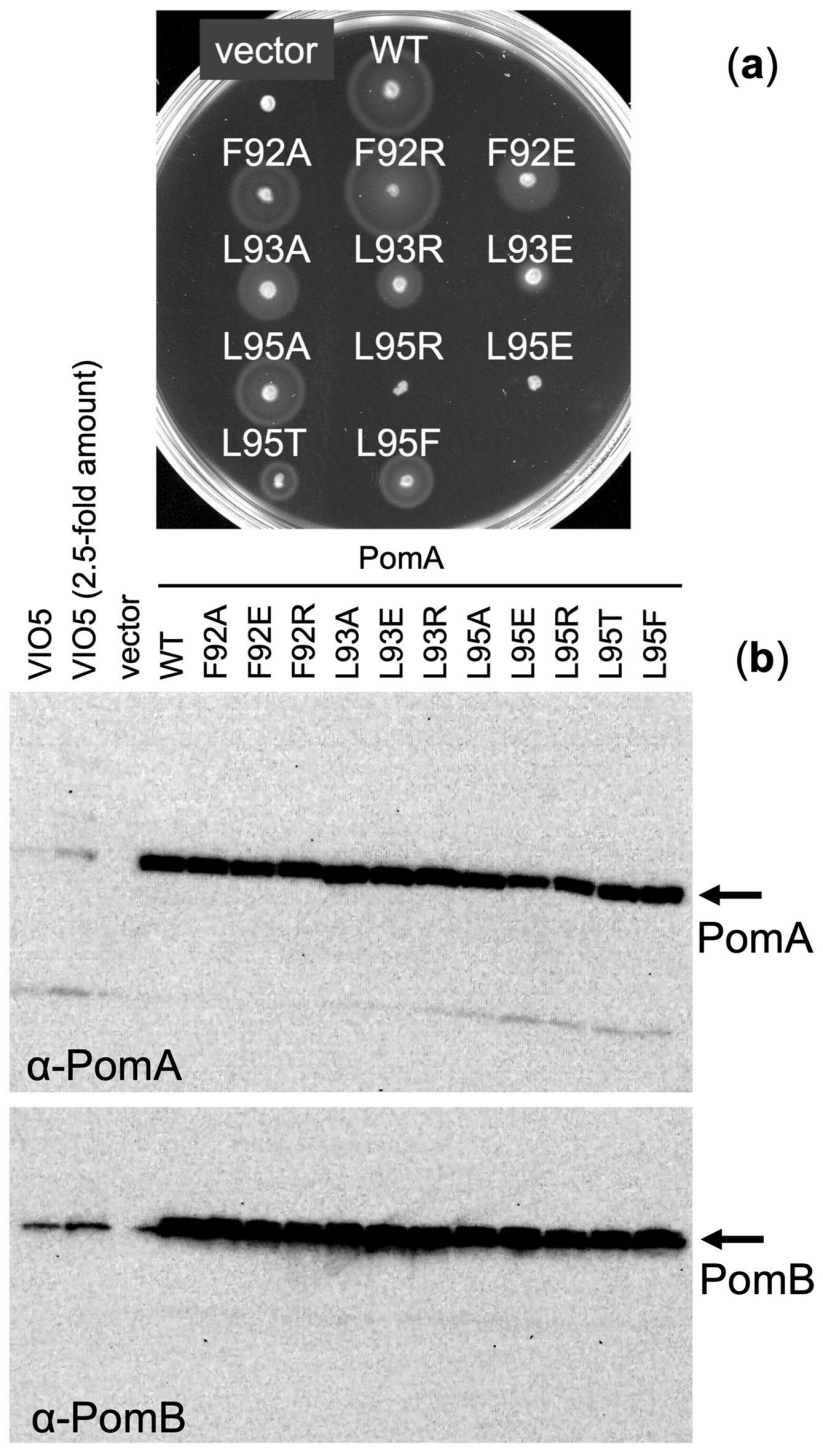
The mutation analysis of PomA by motility and expression. (**a**) Motility assay in a soft-agar plate of *V. alginolyticus* Δ*pomAB* strain NMB191 cells expressing PomA and PomB from the plasmid pHFAB. The cells were inoculated in VPG 0.25% (w/v) agar plate with 0.02% (w/v) arabinose and chloramphenicol at 30 °C for 4 h. (**b**) The proteins of PomA and PomB expressed from plasmid pHFAB in the Δ*pomAB* cells were separated. The proteins of wild-type cells, VIO5, of *V. alginolyticus* were loaded in the left two lanes. The samples for SDS-PAGE were prepared from whole cell lysates. Upper and lower panels showed immunoblot images by using anti-PomA and anti-PomB antibodies, respectively. The regions of interests were presented and the original data for the whole membranes of immunoblot were shown in Fig. S7. The motility assay and immunoblotting were carried out twice.

A previous report showed that F92 and L93 of PomA are located very close to the C-terminal region of FliG in the rotor and suggested that these residues are involved in the interaction between the stator and rotor in addition to conserved charged residues^7^. These two hydrophobic residues are highly conserved among orthologs of stator A subunits, implying functional significance of hydrophobicity at this position (Fig. 1a, Fig. S1). Furthermore, PomA-L95 is also a highly conserved leucine residue (or the similar residue of isoleucine) and is located next to PomA-E96, which is a conserved and important charged residue for the motor function. Thus, we substituted these residues with alanine, glutamate, arginine, phenylalanine or threonine and analyzed motility of cells expressing the mutant PomA proteins. We first examined colony expansion in soft agar plates (Fig. 2a). Alanine or glutamate substitutions of PomA-F92 did not affect motility, but arginine substitution slightly affects motility, suggesting that hydrophobicity at this position is not required for motility. On the other hand, the glutamate-substituted mutant at the position of PomA-L93 severely decreased the size of swimming ring in the soft agar plate, and the arginine-substituted mutant moderately decreased the ring size suggesting that the charged side chain at this position reduces motility. In the substituted mutants at the position of PomA-L95, the alanine and phenylalanine mutants retained motility although it was not as the wild-type level, the threonine mutation moderately reduced the motility, and the arginine and glutamate mutants showed no motility in the soft agar plate. These results suggest that the motility is affected by the charged or hydrophilic side chains at this position.

We examined the protein expression levels of the mutant PomA using immunoblotting for anti-PomA and anti-PomB antibodies (Fig. 2b). The PomA-L95E and L95R mutant proteins were detected at a reduced level than the wild-type PomA produced from plasmid pHFAB induced by 0.02% arabinose. Since the chromosomal endogenous expression level of PomA can hardly detected by the anti-PomA antibody used in this study, we concluded that the mutant proteins expressed from plasmid were sufficent for the function in the *Vibrio* cells (Fig. 2b). This result suggested that the motility defects of the mutants in the soft agar plate were not due to the decrease in the expression level of the mutant proteins.

We next examined the effects of temperature on motility. Cells expressing wild-type PomA, L93E, or L95R were incubated at 20 °C, 30 °C or 40 °C in the soft agar plate (Fig. S2). Wild-type PomA conferred a larger swimming ring at 40 °C than at 30 °C. On the other hand, the L93E and L95R mutants conferred a smaller swimming ring at 40 °C than at 30 °C. At 20 °C, it takes longer time to detect the swimming abilities than at 40 °C and 30 °C probably because of the growth effects. The relative swimming abilities of wild-type PomA and mutant PomA were similar under each temperature condition. These results implied that the mutant PomA protein is more sensitive to high temperatures than the wild-type protein.

### PomA-L93X and L95X mutants conferred a rotationally direction-biased phenotype

**Figure 3:**
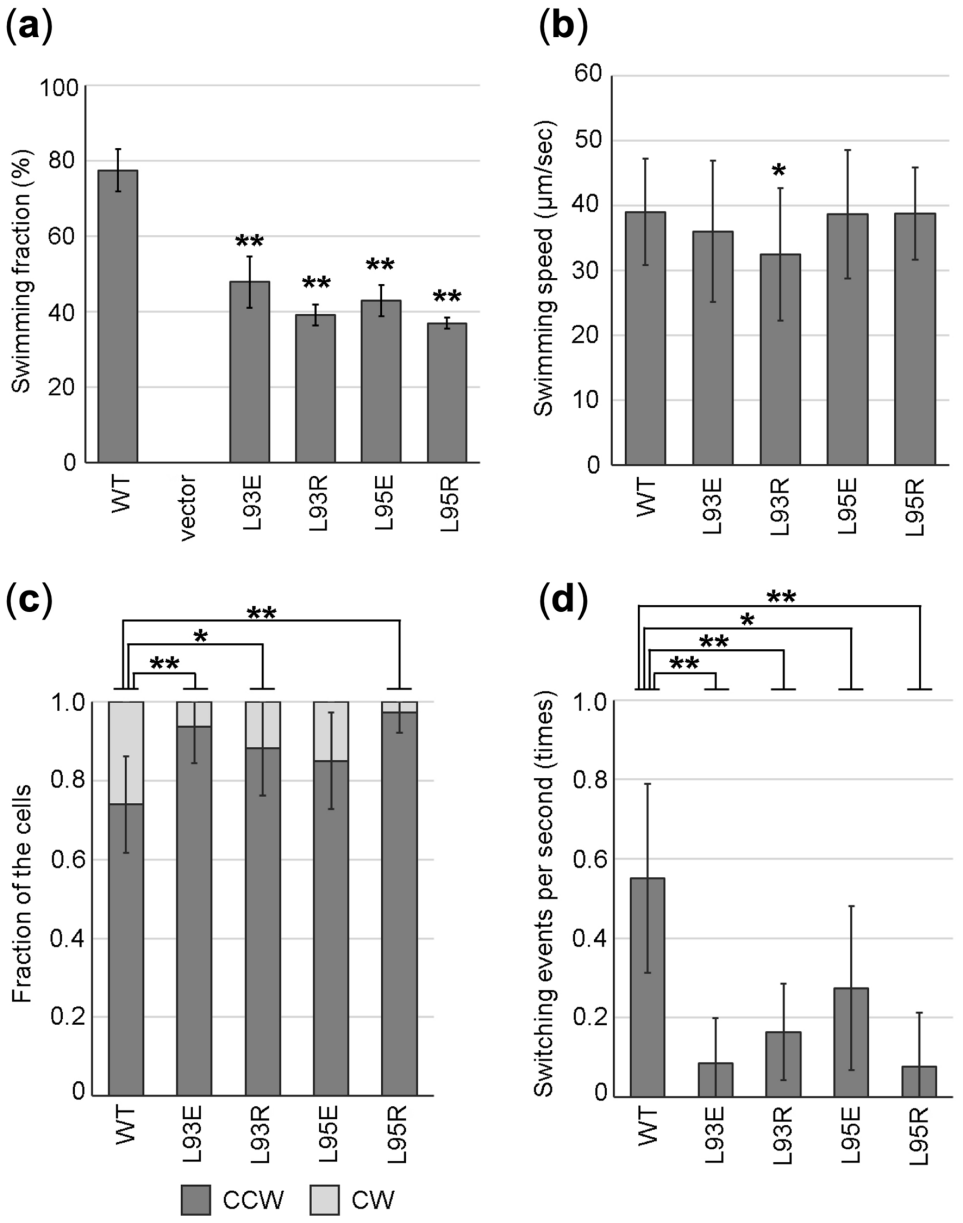
The switching profile in rotation direction of PomA mutants. The PomA and PomB are expressed in *V. alginolyticus* Δ*pomAB* strain NMB191 cells from the plasmid pHFAB, and the mutants were observed by high-intensity dark-field microscopy or by standard dark-field microscopy. (**a**) The cells were video-recorded four times, then swimming fractions of the cells were calculated by dividing the number of the motile cells into the number of the total cells. All experiments were performed three times, and the average and SD of swimming fraction were calculated. (**b**) The cells were video-recorded at 30 frames/s for 5 s, and the swimming speeds of the individual 10 cells measured to calculate the averages and SDs of the swimming speeds. The ratio of CCW/CW rotation (**c**) and the switching frequency (**d**) were calculated from video capturing the 10 s motion of the cells. At least 10 cells were tracked in all the experiments, then the average of the ratio of CCW/CW rotation with SD and the average of switching events with SDs were calculated. Asterisks indicates P < 0.05 (*) and P < 0.01 (**) for wild-type PomA versus the mutants of PomA by the Welch’s *t* test.

**Figure 4:**
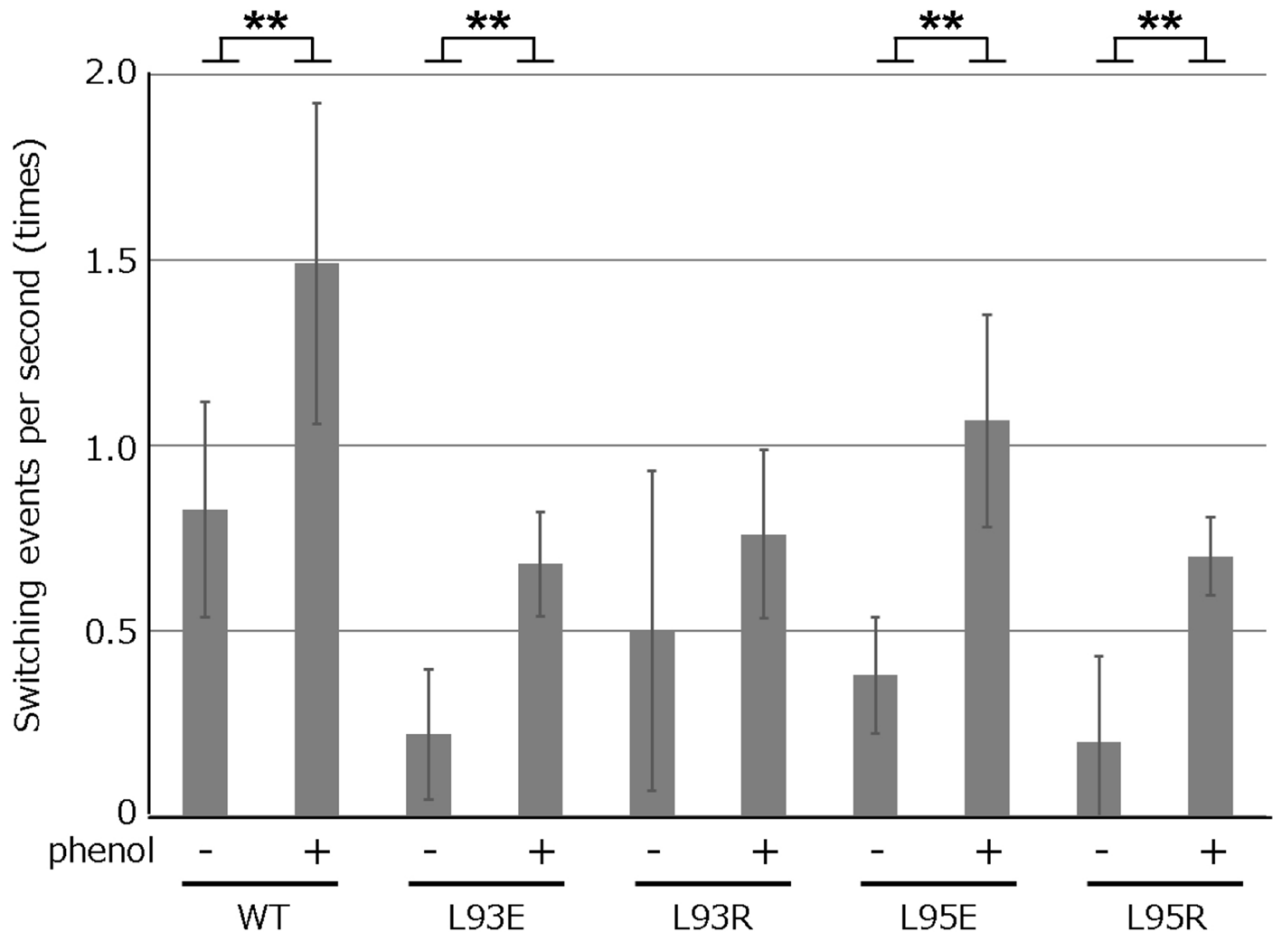
The effect of phenol for the switching of rotational direction. The switching frequencies were calculated similarly to Fig. 3d, from video motion of the cells captured for 10 s, with and without phenol. Asterisks indicates P < 0.01 (**) for without phenol versus with phenol by the Welch’s *t* test.

We next characterized the motility of the cells expressing PomA-L93E, L93R, L95E, or L95R, as these mutants severely affected motility in the soft agar plate (Fig. 2a). We captured cell motion under the microscope, and then analyzed motility properties: motile fraction, swimming speed, switching frequency, and ratio of rotational direction (CCW/CW). These four *pomA* mutants showed a significant decreased motile fraction compared to wild-type PomA (Fig. 3a), suggesting that L93 and L95 may contribute to the stable interacion of stator–rotor in the motor. However, the swimming speed of these *pomA* mutants was not significantly different from that of wild-type PomA (Fig. 3b), indicating that the L93E, L93R, L95E and L95R does not affect torque generation by the flagellar motor. On the other hand, the ratio of the rotational direction of the PomA-L93E, L93R, and L95R mutants, but not of the L95E mutant, significantly biased in the CCW direction compared to that of wild-type PomA (Fig. 3c). Moreover, the switching frequency of all mutants was significantly decreased compared to the wild-type (Fig. 3d). Therefore, these results indicates that these mutations affect the switching event of the flagellar motor. However, the *che* function was not completely lost by these mutations because the response of phenol to the CW rotation of flagella remained active (Fig. 4). Therefore, we found novel mutations in the stator protein conferring the Che^−^ phenotype. This result indicates that the mutations in the stator affect the switching of the rotational direction.

### PomA CCW-biased mutants combined with CW-locked or CW-biased FliG mutant

**Figure 5:**
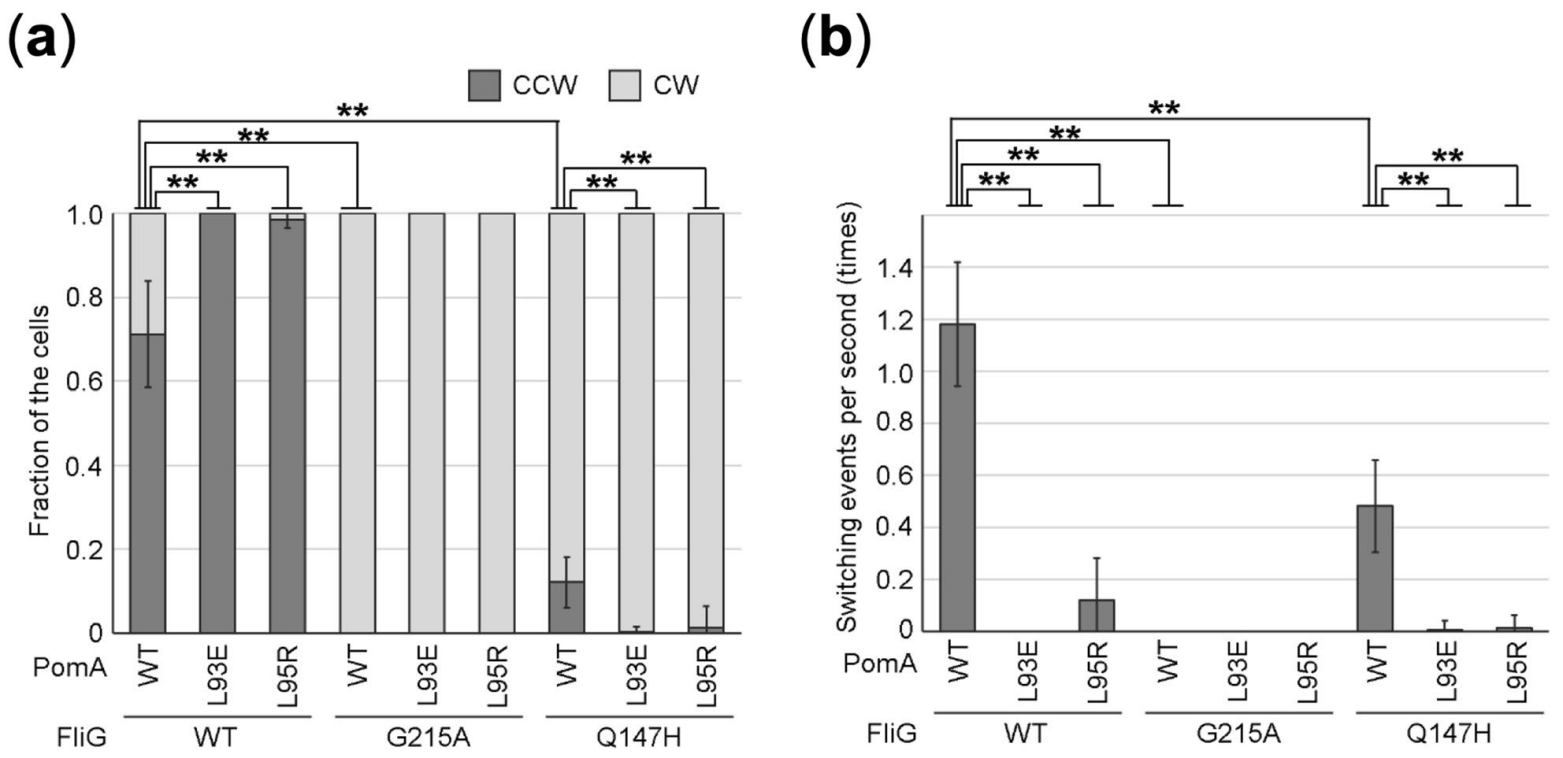
The switching frequency and rotational direction of PomA/FliG double mutants *V. alginolyticus* Δ*pomAB* and *fliG* strain NMB301 cells expressing PomA and PomB from the plasmid pYA303 and FliG from the plasmid pNT1. Those mutants were observed by high-intensity dark-field microscopy. The ratios of CCW/CW rotation (**a**) and the switching frequency (**b**) were calculated from video motion of the cells captured for 10 s. At least five cells were tracked in all the experiments, then averages of the ratios of CCW/CW rotations with SD were calculated. Asterisks indicates P < 0.05 (*) and P < 0.01 (**) for wild-type PomA versus the mutants of PomA by the Welch’s *t* test.

PomA L93 and L95 mutants, as described above, exhibited motility properties biased toward the CCW direction. Next, we examined whether PomA mutations are dominant for switching the rotational direction in combination with CW-locked FliG-G215A mutation or CW-biased FliG-Q147H mutation. When PomA L93E or L95R was combined with FliG-G215A, these two mutations did not showed a CW-locked phenotype of the FliG-G215A mutant (Fig. 5), suggesting that the switching of the rotational direction is predominantly determined by the rotor. The FliG-Q147H mutant showed the CW biased rotation with CCW:CW = 1:9, but a few switching events occurred. In contrast, when PomA-L93E or L95R mutation was combined with FliG-Q147H, these two mutations resulted in more CW-biased rotation and a much lower switching frequency (Fig. 5). This result suggests that the mutation at the position of PomA L93 or L95 biases the rotational direction of the motor by reducing the switching frequency.

## Discussion

The rotational direction of the flagellar motor is mainly controlled by CheY-P and the C-ring proteins, FliG, FliM, and FliN. In this study, we found that mutations in the stator protein PomA affect the switching frequency. These mutations were the residues involved in the stator–rotor interaction (PomA-L93) and the highly conserved hydrophobic residue (PomA-L95) adjacent to the important charged residue (PomA-E96). Since the positions of PomA-L93 and L95 are conserved as hydrophobic residues among various species, hydrophobicity may be important for motor function. In fact, PomA L93E, L93R, L95E, and L95R showed a significant decrease in the ring expansion by swimming in the soft-agar plate, indicating that these mutations affect motor function. Further microscopic observation of these *pomA* mutants revealed that these substitutions result in a significant decrease in the switching frequency, thereby causing a CCW-biased phenotype. This suggests that L93 and L95 of PomA are critical for efficient switching of rotational direction of the flagellar motor. Interestingly, these *pomA* mutations significantly reduced the fraction of swimming cells compared to wild-type cells, suggesting that L93 and L95 contribute to the stable interaction of stator–rotor in the motor. On the other hand, the L93E, L93R, L95E, and L95R mutations did not decrease swimming speed, suggesting that L93 and L95 are not directly involved in the torque generation.

It is thought that the rotational direction of the flagellar motor is determined by the structural change of the C-ring. Cryo-electron tomography has revealed that a diameter of the top of the C-ring expands when the rotational direction is changed from CCW to CW^8,36^. By this structural change of the C-ring, it was hypothesized that the rotor interacts with the inner side of the stator facing the rotor axis during CCW rotation, whereas it interacts with the outer side of the stator during CW rotation (Fig. 1b). Atomic-resolution structures of the stator are represented as a barrel-like shape of the pentamer of the A subunits surrounding the dimer of the B subunits, proposing that the pentamer of the A subunit rotates in the CW direction around the dimer of the B subunits and the rotation is transmitted to the rotor C-ring^5,6^. Recent studies have speculated that stator–rotor interactions resemble a mechanical mechanism, similar to a spur gear^5–8,36^. In other words, the A subunits and the C-ring function like a small gear and a large gear, respectively. The teeth of the small gear (A subunits of the stator) located on the outer side and inner side of the motor engage during CCW rotation and CW rotation, respectively (Fig. 1b). The rotational direction is determined by which side of the stator gear, outer side or inner side, engages according to the change in the size of the large gear (the rotor).

In this study, we propose that the stator also plays some roles in rotational switching of the flagellar motor. The mutations at the position of PomA-L93 or L95 decreased the switching frequency and made CCW bias of the WT motor and CW bias of a CW-biased mutant motor stronger, indicating the suppression of the stator in switching its interaction surface of the rotor ring between the external and internal sides. This hypothesis is supported by the motility property exhibited by a combination of switching frequency-decreased PomA mutation and CW-biased FliG-Q147H mutation. The resultant mutants showed more CW-biased rotation by lowering the switching frequency than the FliG-Q147H mutant. This suggests that the mutation at the position of PomA-L93 or L95 suppresses the shift in the stator position to switch its rotor-interaction side between the inner and outer sides. It has been proposed in a recent report that the increase of force between stator and rotor modulates the binding affinity of the rotor against CheY-P^34^. Based on the proposal, the evidence, that the PomA mutants showed CCW-biased rotation and phenol significantly affected the switching frequency of the flagellar motor, implies that the PomA mutations modulate the force of the stator–rotor interaction, therefore, the binding affinity of the rotor for CheY-P may change.

How are PomA-L93 and L95 involved in switching rotor gears? When PomA-L93 was substituted with charged residues, the affected switching implies that the charged residues may participate in the electrostatic interaction with FliG in the rotor, together with PomA-R88, K89, and E96, which are thought to be the residues involved in the stator–rotor interaction. Because the position of PomA-L95 is located inside the stator structure, the charged residues around this position may change the arrangement of PomA R88 and E96. Interestingly, our previous study showed that PomA R88A/K89A/E96Q/E97Q/E99Q conferred CCW-biased rotation to the motor^39^. These results suggest that the proper number and location of the charged residues, which may define the stator–rotor interaction, compensates proper switching of the rotational direction. Moreover, to achieve the proper electrostatic interaction, the conserved hydrophobic residues may act as effective insulators.

We tried to obtaine swimming mutants in the soft agar plate from the cells expressing PomA-L93E, L95E, or L95R, to identify the factors involved in switching frequency of motor rotation (Figs. S3, S4, S5). Although we had expected that a suppressor mutation is caused in *fliG*, *fliM* or *fliN*, which are the components of the C-ring or rotor, we could not obtained such suppressor mutants. Instead, putative suppressor mutations were caused in genes encoding F_o_F_1_-ATP synthase, a gene encoding ATPase of the Type II secretion system and so on (Table S2). In the previous studies, we have shown that mutations in *uncB*, which encodes the α-subunit of ATP synthase, induced increased motility in soft agar plates^40,41^. The defects in ATP synthase seem to increase the proton gradient generated by the respiratory chain and eventually to increase the sodium-motive force. Therefore, the swimming ring-restored mutants whose mutation was mapped in the component of ATP synthase might allow motility to be restored by increasing the ion motive force, but not by affecting directly the interaction between stator and rotor.

In summary, we provide a genetic evidence that PomA mutations located at a physical interface between stator and rotor inhibit the switching of flagellar motor rotation. Even though the electrostatic interaction becomes either too strong or too weak, shifting the interaction site between the stator and rotor may become difficult. This finding suggests that proper switching of the interaction between the stator and rotor is also important for controlling the rotational direction.

## Materials and methods

### Bacterial strains and plasmids

The bacterial strains and plasmids are listed in Table S1. *E. coli* was cultured in LB broth (1% [w/v] bactotryptone, 0.5% [w/v] yeast extract, 0.5% [w/v] NaCl) at 37 °C. *V. alginolyticus* was cultured in VC broth (0.5% [w/v] polypeptone, 0.5% [w/v] yeast extract, 3% [w/v] NaCl, 0.4% [w/v] K_2_HPO_4_, 0.2% [w/v] glucose], or VPG broth [1% (w/v) polypeptone, 3% (w/v) NaCl, 0.4% (w/v) K_2_HPO_4_, 0.5% (w/v) glycerol] at 30 °C. Chloramphenicol (Cm) was added to a final concentration of 25 µg/mL for *E. coli* and 2.5 µg/mL for *V. alginolyticus*. Ampicillin (Amp) was added at a final concentration of 100 µg/mL for *E. coli*. Kanamycin was added at a final concentration of 50 µg/mL for *E. coli* and 250 µg/mL for *V. alginolyticus*. Arabinose for protein expression from pHFAB was added at a final concentration of 0.02% (w/v) for *V. alginolyticus*.

### Swimming assay in soft agar plates

*V. alginolyticus* NMB191 cells harboring pHFAB were inoculated on VPG 0.25% (w/v) agar plates with 0.02% (w/v) arabinose and 2.5 µg/mL Cm incubated at 30 °C for the desired time.

### Mutagenesis

Site-directed mutagenesis was performed using the QuikChange site-directed mutagenesis method, as described by Agilent Technologies (Santa Clara, USA). Transformation of *V. alginolyticus* by plasmids pHFAB or pYA303 was carried out by electroporation^42^. Transformation of *V. alginolyticus* by plasmid pNT1 was carried out by conjugational transfer from *E. coli* S17-1^43^.

### Measurement of swimming speed and motile fraction

*V. alginolyticus* cells, cultured overnight, were inoculated into fresh VPG broth with antibiotics and incubated by shaking at 30 °C for 4 h. The cells were suspended at a 100-fold dilution in V buffer (50 mM Tris–HCl pH 7.5, 300 mM NaCl, 5 mM MgCl_2_), and then spotted onto glass slides for microscopic observation. The motile fraction was calculated by dividing the number of motile cells by the total number of cells. All experiments were performed three times, and the averages of the motile fraction and standard deviation (SD) were calculated. To measure the swimming speed of an individual cell, the cell was suspended at a 100-fold dilution in V buffer, and serine was added at a final concentration of 5 mM to allow the cell to swim straight. The motion of the cells was captured at 30 frames/s for 5 s under a microscope. The swimming speed of individual 10 cells was measured using ImageJ software (Rasband W.S., Bethesda, USA), and the average swimming speed and SD were calculated.

### Measurement of switching frequency and ratio of CCW/CW rotation

*V. alginolyticus* cells, cultured overnight, were inoculated into fresh VPG broth with antibiotics and incubated by shaking at 30 °C for 4 h. The cells in the 200 μL culture were collected by centrifugation at 2000×*g* for 5 min. The precipitated cells were resuspended in 200 μL of V buffer. The cells were then spotted onto glass slides for high-intensity dark-field microscopic observation. The motion of the cells was captured at 30 frames/s for 10 s under a microscope. The number of frames of either CCW or CW rotation was recorded, and the ratio of CCW/CW rotation and switching events was calculated for 1 s using ImageJ software.

### Whole genome analysis of swimming ring-restored mutants in soft agar plates

The genomic DNA of swimming ring-restored mutants was prepared using a Monarch Genomic DNA Purification Kit (New England Biolabs, Ipswich, USA). The concentration of the purified genomic DNA was adjusted to approximately 50 ng/μL. Genomic DNA libraries were constructed by the tagmentation method on magnetic beads using the Nextera DNA Flex Library Prep kit (Illumina, San Diego, USA) with some modifications. 1 μL of genomic DNA (~ 10 ng) was mixed with 6.5 μL of tagmentation Reaction mix (beads linked transposome 0.5 μL, X5 TAPS buffer 1.5 μL, and miliQ water 4.5 μL; X5 TAPS buffer containing 50 mM TAPS-NaOH, pH = 7.5, 50 mM MgCl_2_, 50% (w/v) DMF), incubate at 55 °C for 25 min and transposases were inactivated by incubation at 80 °C for 3 min. Magnetic beads with tagmented DNA were washed with 100 μL of tagmentation wash buffer (10 mM Tris–HCl pH 8.0, 30 mM NaCl, 0.1% [w/v] Triton X-100) and recovered by setting on a magnetic plate. After discarding the tagmentation wash buffer, 20 μL of PCR reaction mix (KAPA HiFi HS Ready mix; Nippon Genetics) and a set of indexed primers (2.5 μL each) were mixed with the magnetic beads and processed under the following conditions: 72 °C for 5 min, 98 °C for 30 s, (98 °C for 20 s, 62 °C for 15 s, 72 °C for 1 min) × 15 cycles, and at 72 °C for 1 min. Amplified genomic DNA libraries were size-selected around 300–1000 bp using ProNex beads (Promega, Madison, USA), and their sizes were confirmed by the microtip electrophoresis analyzer MultiNA (Shimadzu, Kyoto, Japan). DNA concentration was determined using SYBR Green I (Takara Bio, Kusatsu, Japan). Twenty DNA library samples were pooled to even out the amount of DNA in each library. A mixed library sample was sequenced using HiSeq-X (150 bp PE; Illumina, San Diego, USA), and 20 samples were separated by their index sequences.

### Identification of putative mutation sites using breseq

Genome sequences of the mutants were analyzed using breseq v0.35^44^ with default parameters. For the reference sequence, we used *V. alginolyticus* VIO5 DNA, chromosome 1, complete sequence (accession number: AP022861), and *V. alginolyticus* VIO5 DNA, chromosome 2, complete sequence (accession number: AP022862).

## Supplementary Information

Supplementary Information.
