# Supplementary material for: Mutations in the stator protein PomA affect switching of rotational direction in bacterial flagellar motor
Source: Sci Rep. 2022 Feb 22;12:2979. doi: 10.1038/s41598-022-06947-5 (PMC8863984; doi:10.1038/s41598-022-06947-5)
Supplement: Supplementary file 1 — Supplementary Information. [file 41598_2022_6947_MOESM1_ESM.pdf]

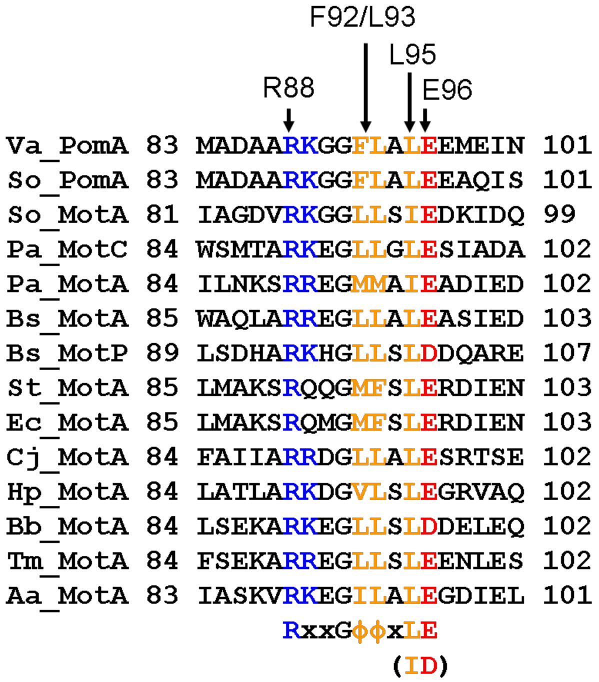

Fig. S1. The putative interaction region between rotor and stator. Alignments of the amino acid sequences of PomA and MotA from various species. The conserved charged residues, important for motor function, were represented in blue (positive) and red (negative). The conserved hydrophobic residues are shown in orange. Abbreviations: Φ, hydrophobic residues; Va, *Vibrio alginolyticus* VIO5; So, *Shewanella oneidensis* MR-1; Pa, *Pseudomonas aeruginosa* PAO1; Bs, *Bacillus subtilis* subsp. *subtilis* 168; St, *Salmonella enterica* subsp. *enterica* serovar *Typhimurium* LT2; Ec, *Escherichia coli* K-12 MG1655; Cj, *Campylobacter jejuni* subsp. *jejuni* NCTC 11168; Hp, *Helicobacter pylori* 26695; Bb, *Borrelia burgdorferi* B31; Tm, *Thermotoga maritima* MSB8; Aa, *Aquifex aeolicus* VF5.

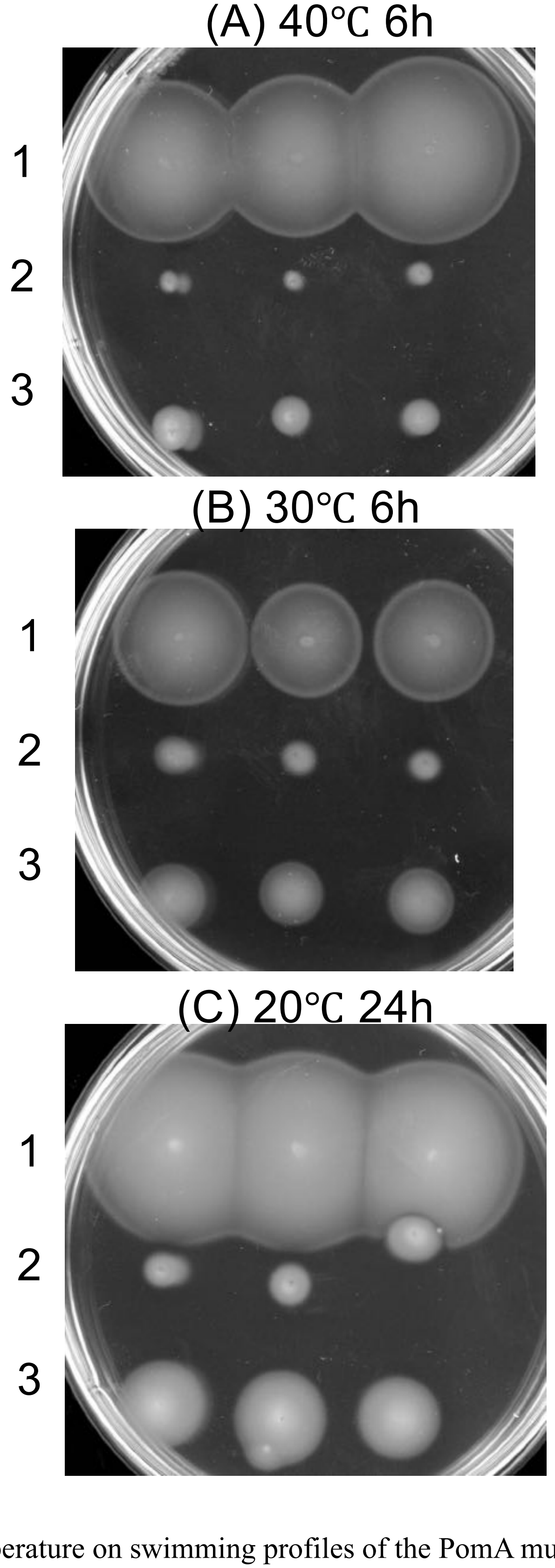

Fig. S2. The effect of temperature on swimming profiles of the PomA mutants. *V. alginolyticus* Δ*pomAB* strain NMB191 cells, expressing PomA and PomB from the plasmid pHFAB (WT: 1) and the mutant plasmids (L95R: 2, L93E: 3), were inoculated in a VPG 0.25% (w/v) agar plate with 0.02% (w/v) arabinose and chloramphenicol at 40°C for 6 h (A), at 30°C for 6 h (B) or at 20°C for 24 h (C). The colonies were inoculated 3 times in line.

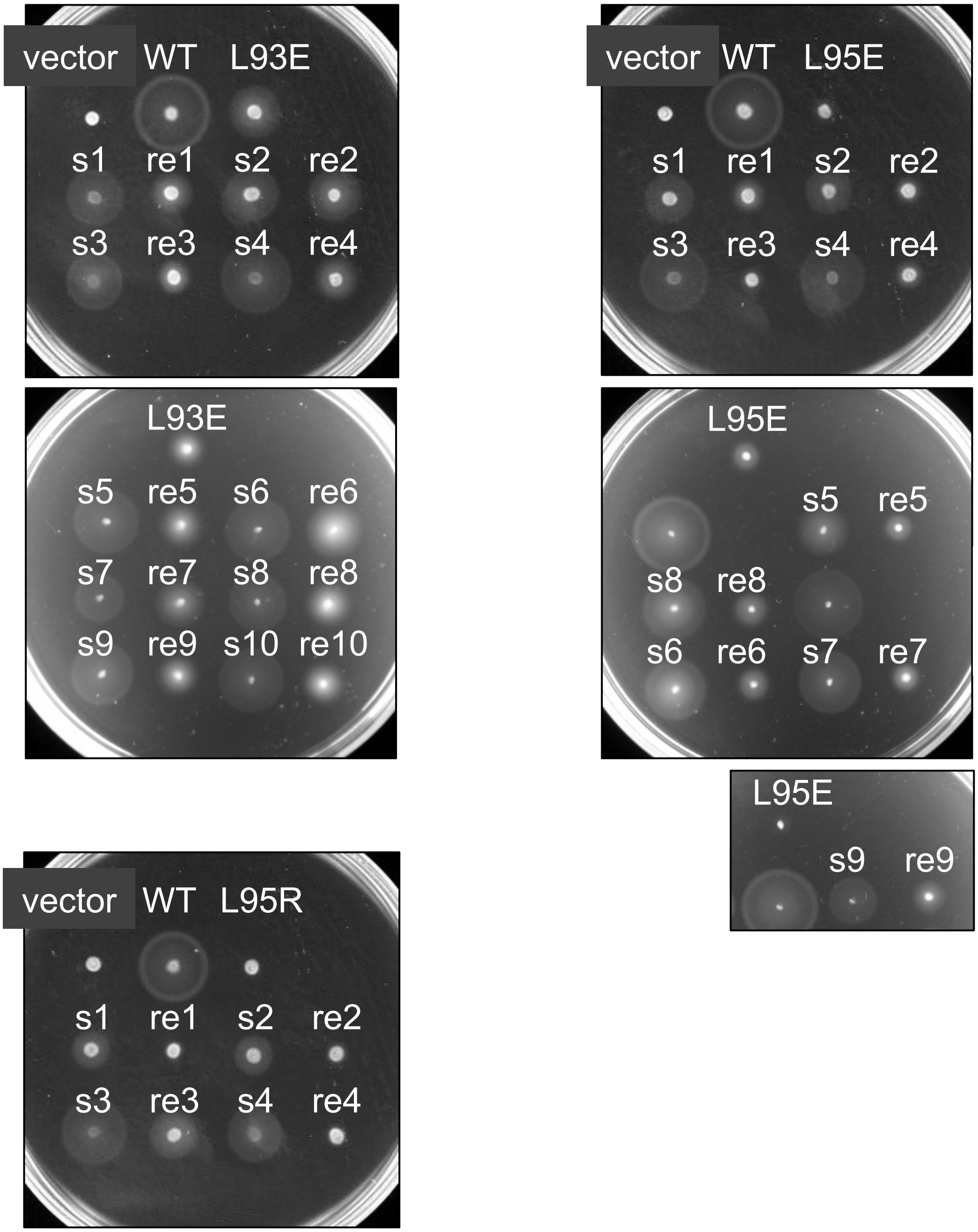

Fig. S3. Motility of the swimming ring-restored mutants in PomA L93E, L95E and L95R. The cells were inoculated in a VPG 0.25% (w/v) agar plate with 0.02% (w/v) arabinose and chloramphenicol at 30 ºC for 4 h. The numbered "s" shows the original swimming ring-restored mutant. The numbered "re" shows the NMB191 cells re-transformed by the plasmid pHFAB extracted from the swimming ring-restored mutants.

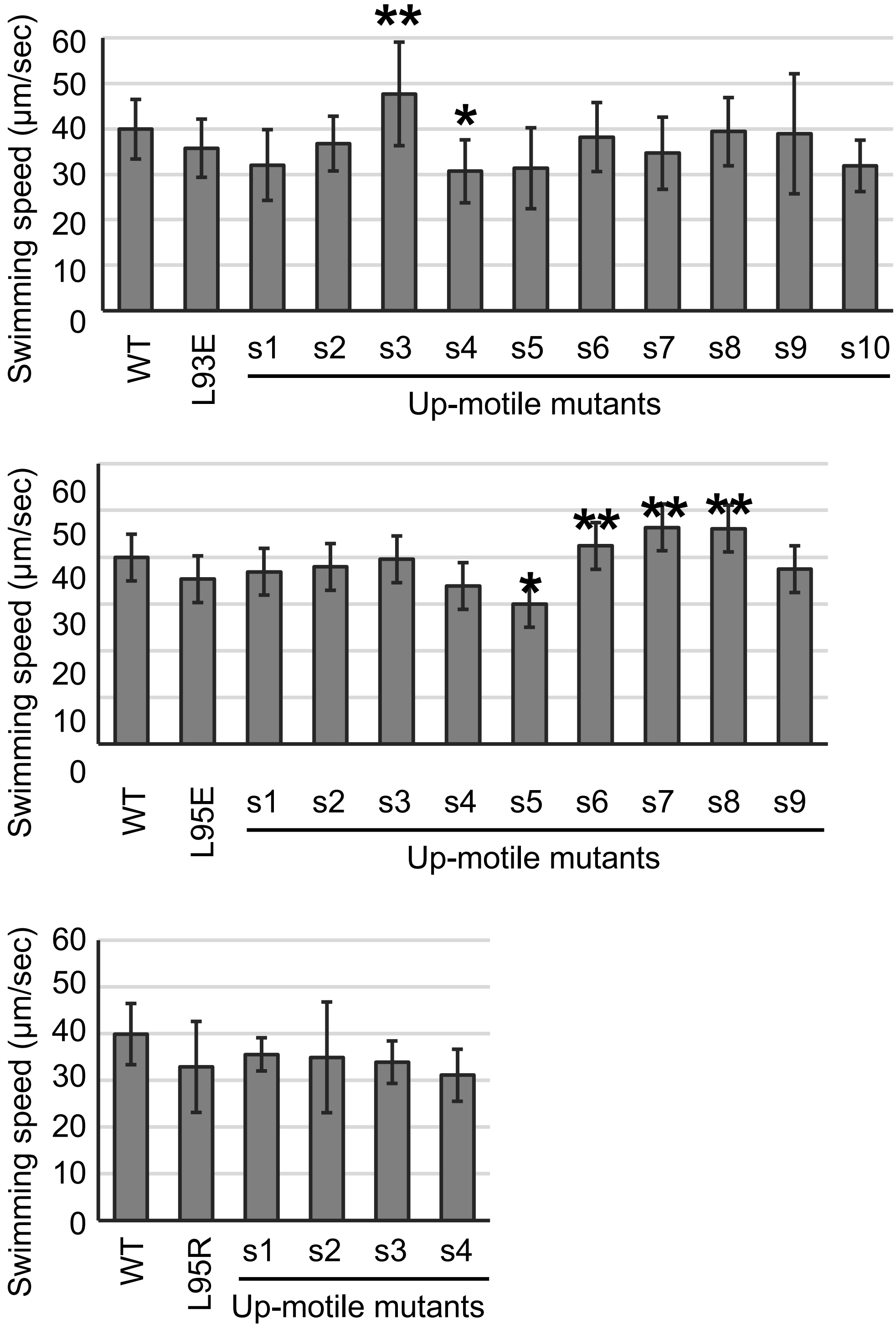

Fig. S4. Swimming speed of the swimming ring-restored mutants. *V. alginolyticus* Δ*pomAB* strain NMB191 cells, expressing PomA and PomB from the plasmid pHFAB, were observed by dark-field microscopy. The cells were video-recorded at 30 frames/s for 5 s, the swimming speed of the individual 10 cells was measured, and then the average of the swimming speed and SD were calculated. Upper, middle and lower panels showed L93E and its swimming ring-restored mutants, L95E and its swimming ring-restored mutants and L95R and its swimming ring-restored mutants, respectively. Asterisks indicated $P < 0.05$ (*) and $P < 0.01$ (**) for wild-type PomA versus the mutants of PomA by the Welch's t-test.

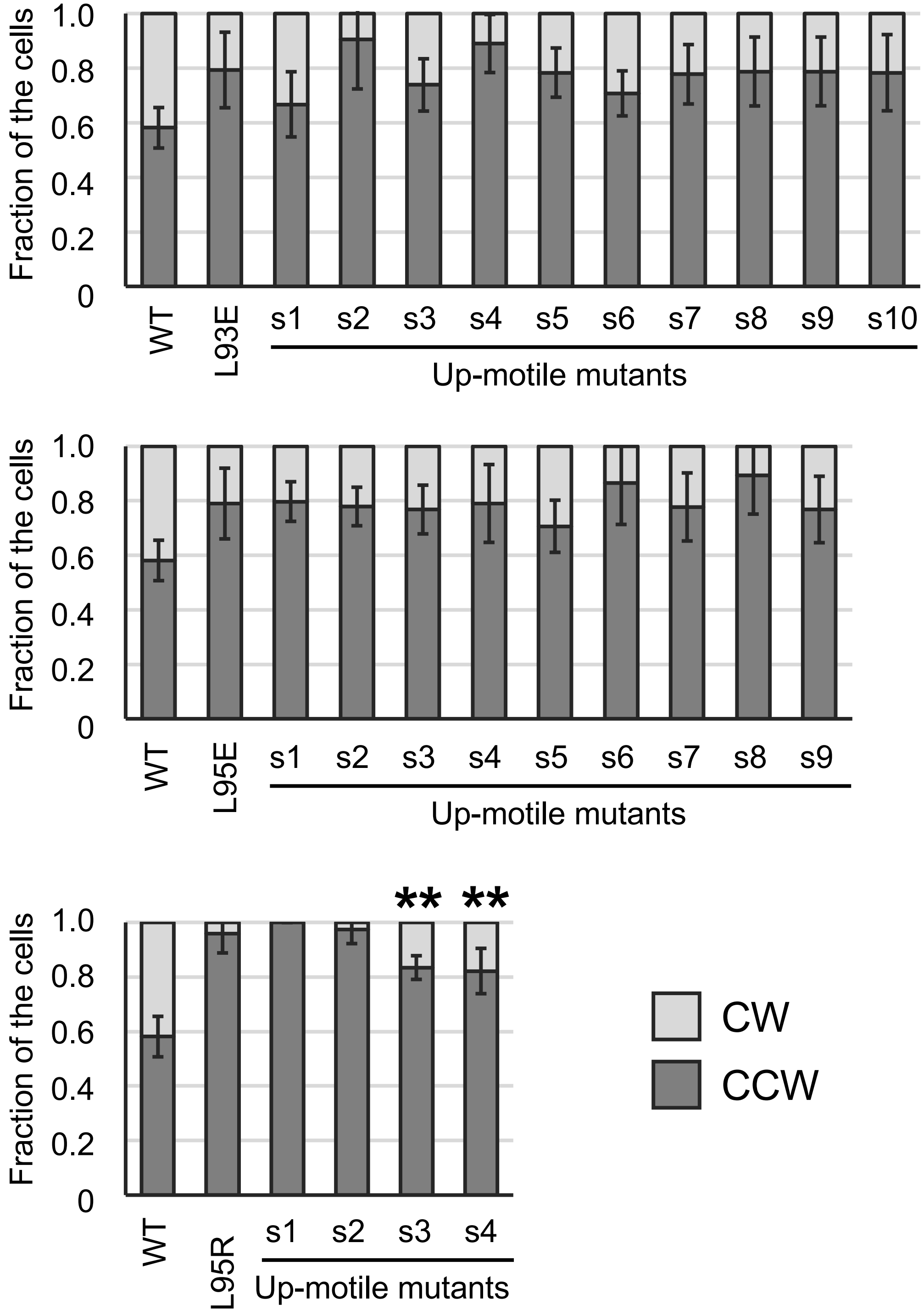

Fig. S5. The profile of the rotation direction of the swimming ring-restored mutants. Cells were observed by high-intensity dark-field microscopy. The ratio of CCW/CW rotation was calculated from video recordings of 10 s. At least 10 cells were tracked in all the experiments, then the average ratio of CCW/CW rotation with SD was calculated. Upper, middle and lower panels showed L93E and its swimming ring-restored mutants, L95E and its swimming ring-restored mutants and L95R and its swimming ring-restored mutants, respectively. Asterisks indicated $P < 0.05$ (*) and $P < 0.01$ (**) for wild-type PomA versus the mutants of PomA by the Welch's t-test.

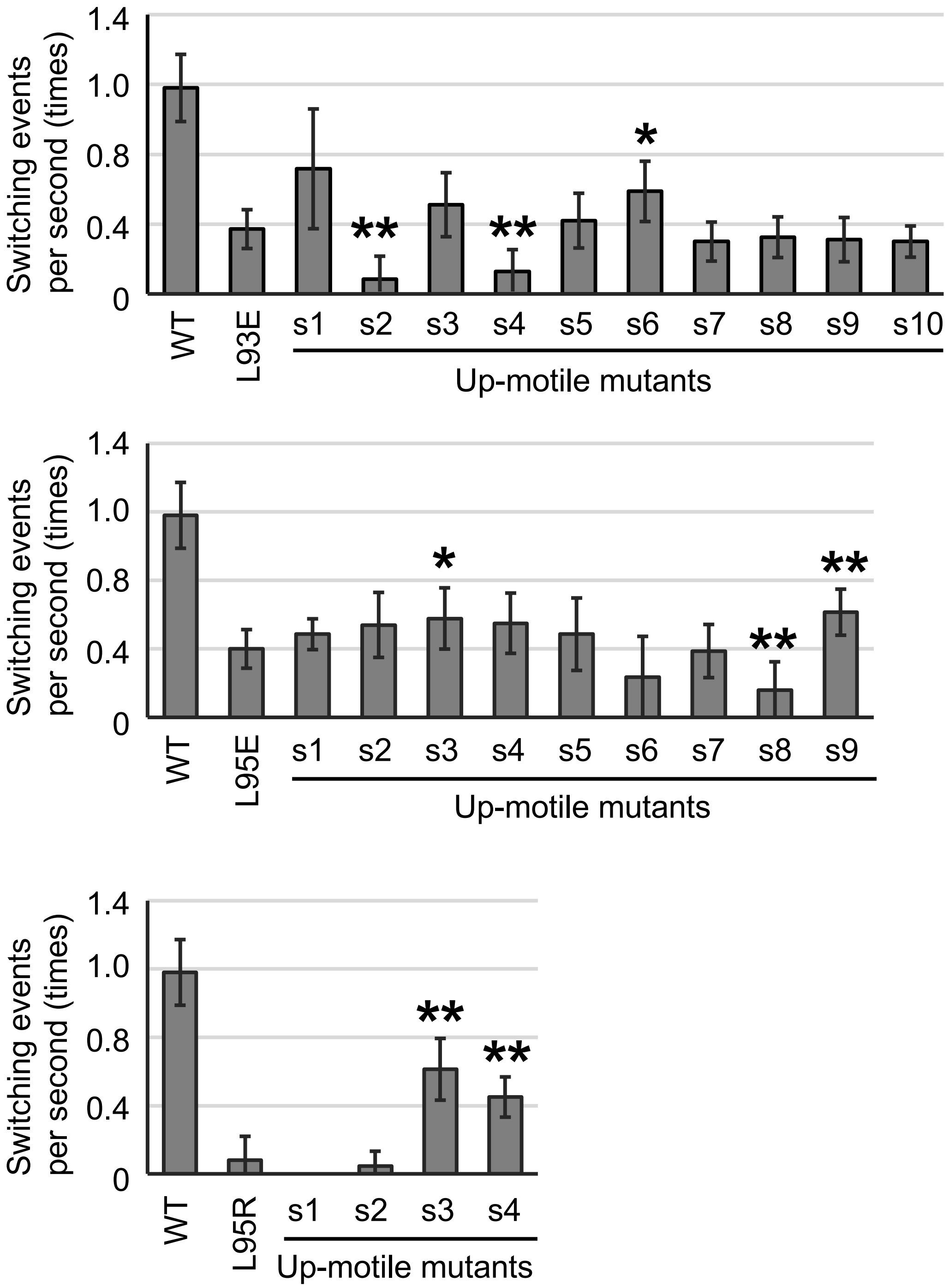

Fig. S6. The profile of the switching frequency of the swimming ring-restored mutants. Cells were observed by high-intensity dark-field microscopy. The switching frequency were calculated from video motion of the cells captured for 10 sec. At least 10 cells were tracked in all the experiments, then average of switching events with standard deviation (SD) were calculated. Upper, middle and lower panels showed L93E and its swimming ring-restored mutants, L95E and its swimming ring-restored mutants and L95R and its swimming ring-restored mutants, respectively. Asterisks indicated $P < 0.05$ (*) and $P < 0.01$ (**) for wild-type PomA

(A) (B)

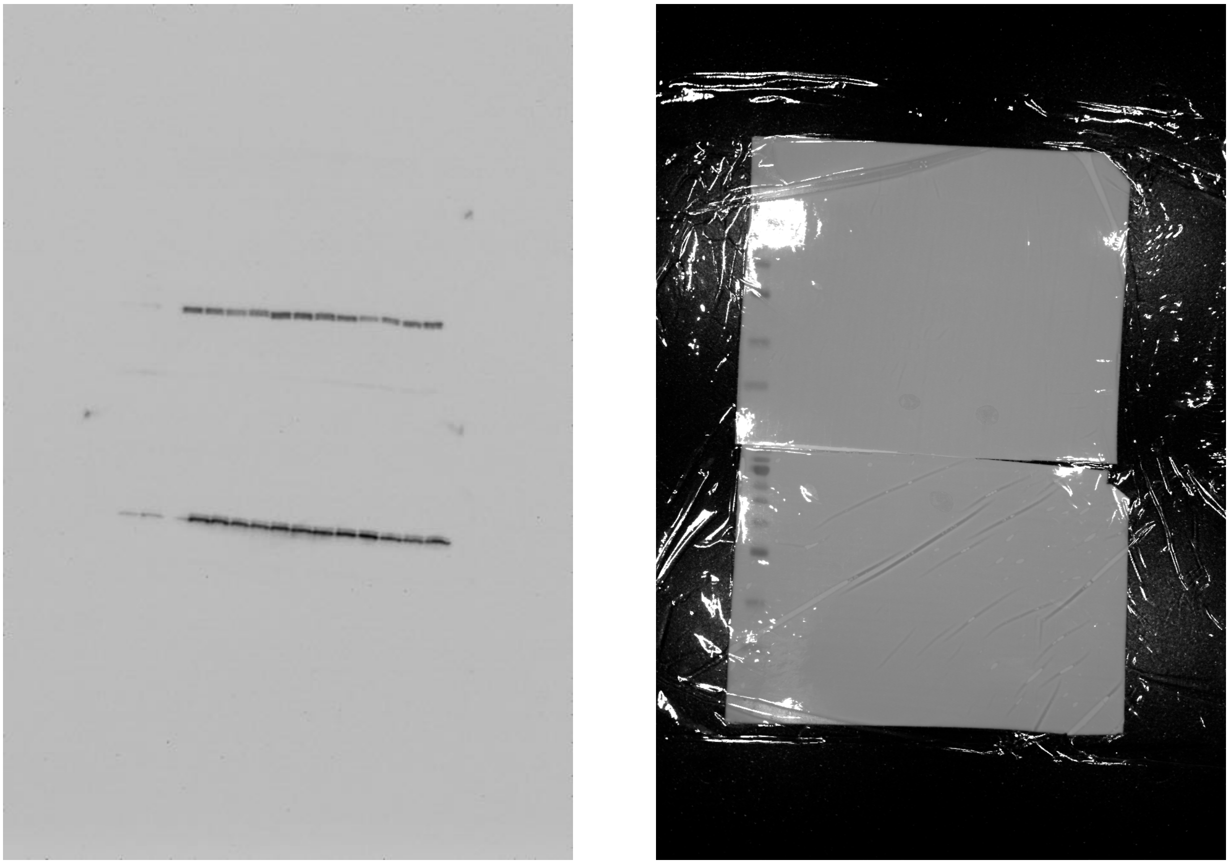

Fig. S7. The entire images of the original blots for Fig. 2B. The proteins were separated by SDS-PAGE and transferred to the membranes. Upper and lower membranes showed the immunoblot images by using anti-PomA and anti-PomB antibodies, respectively (A) or the plain photo images of the membranes, respectively (B). The regions of interests were shown in Fig. 2B.

Table S1. Strains and plasmids used in this study.

Strain or plasmid	Genotype or description	Reference or source
Vibrio alginolyticus		
VIO5	VIK4 *laf* (Rif$^+$ Pof$^+$ Laf$^-$)	(1)
NMB191	VIO5 Δ*pomAB* (Rif^r Pof$^+$ Laf$^-$ Mot$^-$)	(2)
NMB301	NMB191 Δ*fliG* (Rif^r Pof$^+$ Laf$^-$ Mot$^-$ FliG$^-$)	(3)
Escherichia coli		
DH5α	Recipient for cloning	(4)
S17-1	Recipient for conjugation of pMMB206	(5)
**Plasmid**		
pBAD33	Cm^r, P_{BAD}	(6)
pHFAB	*pomA* and *pomB* in pBAD33	(7)
pSU41	Km^r, P_{lac}	(8)
pYA303	*pomA* and *pomB* in pSU41	(9)
pMMB206	Cm^r, P_{tac} P_{lac}UV5	(10)
pNT1	*fliG* in pMMB206	(3)

Rif^r, rifampin resistant; Cm^r, chloramphenicol resistant; Pof$^+$, normal polar flagellar formation; Laf$^-$, defective in lateral flagellar formation; Mot$^-$, motility defect; P_{BAD}, arabinose promoter; MCS, multiple cloning sites.

(1) J Bacteriol 178:2409-2415; (2)J Bacteriol 181:5103-5106; (3) J Bacteriol 196:1377-8; (4) Proc Natl Acad Sci U S A 87:4645-9; (5) Nat Biotechnol 1:784-791; (6) J Bacteriol 177:4121-4130; (7) J Mol Biol 351:707-17; (8) Gene 102:75-78; (9) Microbiology 145:1759-1767; (10) Gene 97:39-47.

Table S2. Mutation positions in the up-motile mutants

	Gene	Description	Position	Mutation	Annotation
L93Es1	*[atpB]–[VagVIO5_30100]*	ATP synthase subunit a	Chr.1: 3,375,252	Δ132 bp	
L93Es2					
L93Es3	*VagVIO5_09470*	porin	Chr.1: 1,063,033	+G	Coding (946/1032)
	atpA	ATP synthase subunit alpha	Chr.1: 3,372,952	+TG	coding (124/1542 nt)
L93Es5	*atpF*	ATP synthase subunit b	Chr.1: 3,373,873	$(CTTGTTCG)_{2\to1}$	coding (228 235/471 nt)
L93Es6	*VagVIO5_15100*	V10 pilin	Chr.1: 1,680,314	G→T	G128C (GGC→TGC)
	atpB	ATP synthase subunit a	Chr.1: 3,374,986	Δ1 bp	coding (321/813 nt)
L93Es7	*atpC*	ATP synthase epsilon chain	Chr.1: 3,368,946	Δ78 bp	coding (143-220/423 nt)
	atpA	ATP synthase subunit alpha	Chr.1: 3,372,646	G→A	Q144* (CAA→TAA)
L93Es8	*ftsH*	ATP-dependent zinc metalloprotease FtsH	Chr.1: 2,645,924	C→A	E665* (GAA→TAA)
	atpF	ATP synthase subunit b	Chr.1: 3,373,873	$(CTTGTTCG)_{2\to1}$	coding (228-235/471 nt)
	rafA	alpha-galactosidase	Chr.1: 3,389,845	C→T	G533S (GGC→AGC)
L93Es9	*atpF*	ATP synthase subunit b	Chr.1: 3,373,873	$(CTTGTTCG)_{2\to1}$	coding (228-235/471 nt)
L93Es10	*atpA*	ATP synthase subunit alpha	Chr.1: 3,372,761	Δ1 bp	coding (315/1542 nt)
L95Es1	*VagVIO5_01130*	type II secretion system protein GspE	Chr.1: 139,273	Δ15 bp	Coding (199-213/1503 nt)
L95Es2	*VagVIO5_01130*	type II secretion system protein GspE	Chr.1: 139,273	Δ15 bp	Coding (199-213/1503 nt)
L95Es3	*VagVIO5_01130*	type II secretion system protein GspE	Chr.1: 143,278	+T	coding (365/390 nt)
L95Es4	*VagVIO5_02130 , VagVIO5_02140*	hypothetical protein	Chr.1: 245,400	$(A)_{8\to7}$	coding (989/993 nt) coding (46/555 nt)
	VagVIO5_07700	PTS N-acetylmuramic acid transporter subunit IIBC	Chr.1: 860,215	C→A	G506G (GGC→GGA)
	VagVIO5_20480	chemotaxis protein CheW	Chr.1: 2,289,408	$(CCTCTG)_{9\to8}$	coding (131-136/1053 nt)
L95Es5	*VagVIO5_20180*	beta-ketoacyl-[acyl-carrier-protein] synthase I	Chr.1: 2,257,753	Δ18 bp	coding (754-771/1212 nt)
	fabZ	3 hydroxyacyl [acyl carrier protein] dehydratase FabZ	Chr.1: 2,370,655	G→A	P107S (CCG→TCG)
L95Es6	*VagVIO5_06350*	phosphatidylglycerophosphatase A	Chr.1: 716,371	T→G	D79E (GAT→GAG)
	tkt2	transketolase 2	Chr.2: 515,274	T→C	A533A (GCA→GCG)
			Chr.2: 515,279	A→C	L532V (TTA→GTA)
			Chr.2: 515,286	C→A	T529T (ACG→ACT)
			Chr.2: 515,287	+C	coding (1586/1992 nt)
			Chr.2: 515,289	A→G	D528D (GAT→GAC)
			Chr.2: 515,291	Δ1 bp	coding (1582/1992 nt)
			Chr.2: 515,295	C→A	E526D (GAG→GAT)
			Chr.2: 515,309	G→A	L522L (CTA→TTA)
L95Es7	*VagVIO5_06350*	phosphatidylglycerophosphatase A	Chr.1: 716,193	G→T	G20V (GGT→GTT)
	VagVIO5_37760, VagVIO5_37770	hypothetical protein/ABC transporter ATP-binding protein	Chr.2: 824,651	G→A	(-310/+193)
			Chr.2: 824,658	T→A	(-317/+186)
			Chr.2: 824,667	T→C	(-326/+177)
			Chr.2: 824,680	T→G	(-339/+164)
			Chr.2: 824,684	T→A	(-343/+160)
			Chr.2: 824,703	C→T	(-362/+141)
			Chr.2: 824,706	T→A	(-365/+138)
			Chr.2: 824,713	4 bp→G	(-372/+128)
L95Rs1	*VagVIO5_01130*	type II secretion system protein GspE	Chr.1: 139,513	Δ1 bp	coding (439/1503 nt)
	VagVIO5_07570	UPF0721 transmembrane protein	Chr.1: 844,041	C→T	L46L (TTG→TTA)
L95Rs2	*VagVIO5_01130*	type II secretion system protein GspE	Chr.1: 139,513	Δ1 bp	coding (439/1503 nt
L95Rs3	*atpD*	ATP synthase subunit beta	Chr.1: 3,369,971	Δ1 bp	coding (617/1404 nt)
L95Rs4	*atpB*	ATP synthase subunit a	Chr.1: 3,374,583	G→C	H242D (CAT→GAT)
